# AFLPs Reveal Different Population Genetic Structure under Contrasting Environments in the Marine Snail *Nucella lapillus* L.

**DOI:** 10.1371/journal.pone.0049776

**Published:** 2012-11-21

**Authors:** Belén Carro, María Quintela, José Miguel Ruiz, Rodolfo Barreiro

**Affiliations:** Department of Animal Biology, Plant Biology and Ecology, Faculty of Science, University of A Coruña, A Coruña, Spain; Institute of Marine Research, Norway

## Abstract

Dispersal has received growing attention in marine ecology, particularly since evidence obtained with up-to-date techniques challenged the traditional view. The dogwhelk *Nucella lapillus* L., a sedentary gastropod with direct development, is a good example: dispersal was traditionally assumed to be limited until studies with microsatellites disputed this idea. To shed some light on this controversy, the genetic structure of dogwhelk populations in northwest Spain was investigated with highly polymorphic AFLP markers giving special attention to the influence of hydrodynamic stress. In agreement with the expectations for a poor disperser, our results show a significant genetic structure at regional (<200 km) and areal scales (<15 km). However, the spatial genetic structure varied with wave-exposure in the present case study: IBD was evident under sheltered conditions but absent from the exposed area where genetic differentiation was stronger. Our results provide evidence that differences in wave-exposure can exert a detectable influence on the genetic structure of coastal organisms, even in species without a planktonic larva.

## Introduction

The quantification of dispersal ability and connectivity between populations is a major challenge in marine ecology with implications for the conservation and management of coastal environments [1], [2]. Dispersal determines where a species can be found and also its demographic structure (continuous or patchy, stable or unstable) [3], [4]. Above all, dispersal supports the gene flow that acts either promoting evolution by transmitting successful alleles or constraining evolution by establishing homogeneity that limits the action of natural selection [3]. The type of development was long thought to be crucial for the final population structure of many species due to its direct relationship with dispersal ability [5]. In marine organisms, populations with a long meroplanktonic phase (weeks, months) are expected to have large gene flow even at extensive spatial scales [6]. This view has lately been challenged with the discovery that the actual dispersal distance of some species could be much smaller than previously thought due to the action of both physical (currents, habitat factors) and behavioral mechanisms [7]–[18].

Estimating dispersal is challenging. Direct measures are only possible in few species [19] and some of them can eventually be misleading [3]. Thus, it is necessary to resort to alternative procedures [20]. Among the latter, the genetic analysis of adult populations allows an indirect inference of dispersal without having to follow specific individuals. Besides, genetic data avoid uncertainties about the final destination of migrants and provide a time-integrated overview of dispersal patterns [21]. Nevertheless, the genetic approach is not free from limitations. An observed genetic structure can sometimes be explained as a consequence of different processes: gene flow, local selection, or even recruitment pattern [5], [22], [23]. Moreover, genetic estimates are often difficult to interpret in ecological terms, although recent developments in data analyses have opened the way to estimate dispersal at ecologically relevant time scales (i.e. events occurring in a year or a few years) under certain circumstances [24]. In spite of these limitations, the lack of alternatives makes genetic estimates the only available option to get at least a rough idea of dispersal in many marine organisms.

The dogwhelk *Nucella lapillus* illustrates the uncertainties surrounding the assessment of dispersal in marine organisms. This snail is the most common neogastropod in Eastern Atlantic intertidal rocky shores ranging from south Portugal to northern Russia, and it even reaches the northern west Atlantic coast of North America [25]. Since the mid-1980s, this species has been intensively studied due its high sensitivity to tributyltin (TBT), a biocide present in anti-fouling paints that disrupts hormonal homeostasis in female dogwhelks causing imposex; i.e. a superimposition of male sexual organs. At its highest expression, imposex results in a blockage of the oviduct that renders the female functionally sterile [26], [27]. TBT resulted in the local extermination of *N. lapillus* in many areas throughout its European range (reviewed in Gibbs and Bryan [28]). Presumably, the demographic consequences of female sterility were exacerbated by the fact that juvenile dispersal, and thereby the arrival of recruits from distant populations, is limited in species with direct development [25], [29]. Moreover, although there are no definitive data on adult mobility and dispersal, traditional view is that adults probably spend their entire lives in the same area, and earlier studies found that both *N. lapillus* and its Pacific congener *N. emarginata* move only a few meters in time periods as long as one year [25], [30]. On the other hand, dogwhelks are rocky dwellers and their populations are often confined to enclaves separated by stretches of coast unsuitable for the species (e.g. soft sediment). Limited dispersal together with a fragmented habitat led to anticipate that, once TBT pollution declines, dogwhelks will be little able to recolonize sites from where they had formerly disappeared. Instead, studies using microsatellites have concluded that dogwhelks must disperse more than anticipated to explain the rapid recovery of genetic diversity in sites recolonized after the ban on TBT paints [31], [32].

Here, we investigated the diversity and genetic structure of populations of *N. lapillus* in Galicia (NW Spain) to obtain indirect inferences about its dispersal ability. Sampling was designed to investigate the influence of wave exposure on genetic structure. We hypothesized that different hydrodynamic conditions could modify the dispersal capacity of the organisms with an impact on the resulting genetic structure. With this aim we sampled two different areas belonging to contrasting hydrodynamic environments.

AFLPs were chosen as molecular markers because of high reproducibility and potential to comprehensively scan the genotype [33]–[35]. Besides, AFLP markers can be more efficient than microsatellite loci in systems characterized by weak population structure [36].

## Materials and Methods

### Sampling and DNA Extraction

**Figure 1:**
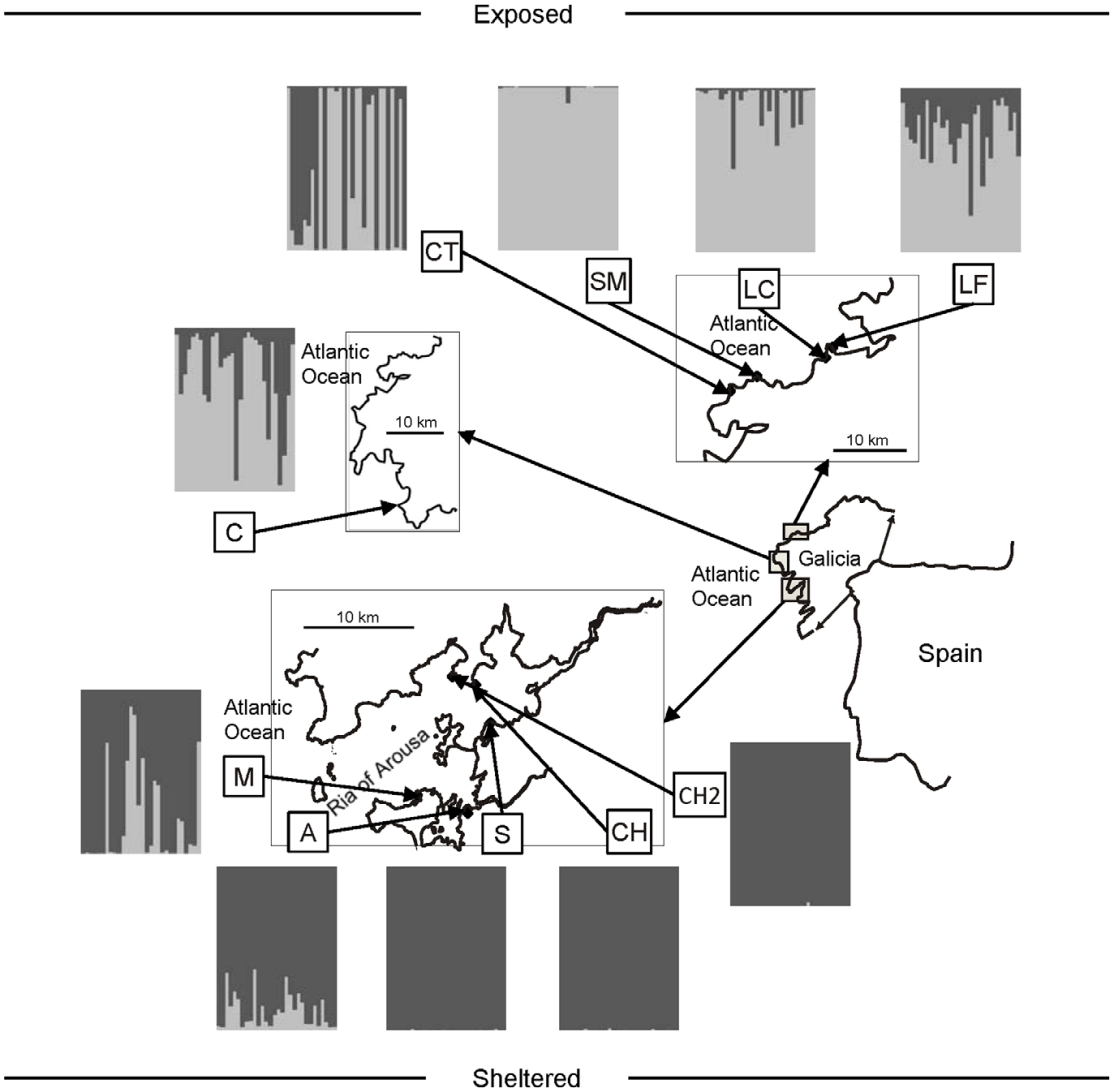
Map combining *Nucella lapillus* sample locations and STRUCTURE output. Histograms are the STRUCTURE output; each individual is represented by a vertical line divided into segments of different color that represent the two clusters detected with a Bayesian approach.

Ten samples consisting of 30 individuals each (15 males and 15 females) were collected in Galicia (NW Spain): five from sites scattered along a exposed stretch of coast (Laxe-F, LF; Laxe-C, LC; Santa Mariña, SM; Cabo Tosto, CT; Caldebarcos, C); and five from locations in a sheltered stretch, all of them within a single ria (*i.e*. a drowned river valley) (Chazo2, CH2; Chazo, CH; As Sinas, S; Isla de Arousa, A; Meloxo, M) (Figure 1). Importantly, sites were selected so that the range of distances among locations (from 400 m to 15 km) was analogous within both areas. The main aim of the study was to compare a sheltered and an exposed area in terms of their population structure (i.e. genetic differentiation between sites, IBD). Therefore, sites within each area were not intended to be considered true independent replicates.

Only subadults, identified by their shell traits [25], were sampled in an effort to analyze individuals from a single cohort [37]. Also, within each site, individuals were collected from the smallest possible area to prevent mixing different breeding groups that could lead to a heterozygosity reduction due to Wahlund effect as seen in other studies with *N. lapillus* [38], [39]. No specific permits were required for the described field studies. Also, no specific permissions were required for these locations/activities, none of the locations was privately-owned or protected in any way, and field studies did not involve endangered or protected species. Shells were opened with a vice and individuals were sexed under the stereomicroscope. Foot and mantle tissue were stored in 96° ethanol at 12°C. To avoid cross contamination, each individual was dissected using disposable tools and/or flame sterilized material. Genomic DNA was extracted from some 3 mg (dry weight) mantle tissue using Qiagen DNeasy tissue kit following manufacturer instructions and stored in TE at −20°C.

### Primer Selection

We carried out a multistage study to select AFLP primer combinations that produced polymorphic, reproducible, and easily scorable patterns. In a first stage, 24 combinations were tested on six individuals from two sites (sheltered and exposed coast, respectively). The 11 combinations that produced the most interpretable banding profiles were then re-tested on 24 individuals from six sites (four sites from the sheltered coast area and two sites from the exposed one; four individuals per site). The six combinations yielding the highest levels of polymorphism were thus retained and tested again on new, independent DNA extractions of the same 24 individuals to assess reproducibility (see primer sequences in Table S1 in Supporting Information).

### AFLP Reactions

AFLP reactions were performed following the protocol of Vos et al. [33] with minor modifications. Briefly, DNA extractions from each individual were diluted in TE buffer to a final concentration of 24–35 ngµl^−1^ [35]; and 10 µl of diluted DNA were restricted with 2.5 units of *EcoRI* and *Tru1I* in a total volume of 20 µl containing 2X Tango buffer (Fermentas). After incubation, the product was added to 6 µl of a ligation solution containing 0.43 µM *EcoRI*-adapters (5′-CTCGTAGACTGCGTACC-3′ and 5′-AATTGGTACGCAGTCTAC-3′), 4.3 µM *Tru1I*-adapters (5′-GACGATGAGTCCTGAG-3′ and 5′-TACTCAGGACTCAT-3′), 0.52 units of T_4_ ligase (Fermentas) and 10X ligation buffer. Ligation products were diluted 10-fold in Milli-Q H_2_O (Millipore Co.) and 10 µl were used for a preselective amplification with 0.3 µM *EcoRI*-primer with a T selective nucleotide (5′-GACTGCGTACCAATTC+T-3′), 0.3 µM *Tru1I*-primer with a C selective nucleotide (5′-GATGAGTCCTGAGTAA+C-3′), 2.5 mM MgCl_2_, PCR buffer, 0.04 µgµl^−1^ BSA, 0.2 µM dNTPs and 0.04 units of *AmpliTaq* polymerase (Applied Biosystems). Ten µl from the 10-fold diluted product of the preamplification were finally used for selective amplifications with 0.6 µM of each *EcoRI* and *Tru1I* primers, both designed with three extra selective nucleotides added to the 3′ end (*EcoRI*/*Tru1I*: TAG/CGT, TAG/CAC, TAG/CTG, TCT/CGT, TCT/CAC and TCT/CTA), 0.8 µM dNTPs, 2.5 mM MgCl_2,_ 0.04 µgµl^−1^ BSA, PCR buffer, and 0.4 units of *AmpliTaq Gold* polymerase (Applied Biosystems). Preamplification was performed immediately after ligation whereas the products of the other reactions were kept overnight at −20°C. PCR reactions were performed in a Hybaid thermocycler model PxE. The 5′ end of the selective E-primers was labeled with FAM or NED fluorochromes.

Negative DNA extraction controls were regularly included to screen for cross contamination. Furthermore, DNA extractions of 10% of individuals were duplicated on different dates and run blindly along the other extracts to avoid bias scoring during reproducibility tests [40]. The estimated genotyping error (1.6%, error rate by primer combination: 1.1–1.9%) was consistent with former studies [40]; and none of the individual loci exceeded the maximum acceptable error rate (0.1) recommended by Bonin et al. [41]. Samples, negative controls, and replicates were randomly distributed in PCR plates. Reactants were always mixed in a laminar flow cabin; DNA and PCR product solutions were always added using filter tips to minimize the risk of cross contamination.

PCR fragments were separated on a 3130×l Genetic Analyzer (Applied Biosystems). Fingerprint patterns were processed with the software GeneMarker v1.70 (Softgenetics) using the options suggested for AFLP and following common recommendations for these markers [42], [43]. Results were translated into a binary matrix of presence/absence of each band. Binary data from the six primer combinations were pooled to obtain a multilocus phenotype for each individual.

### Data Analysis

Allele frequencies were estimated using the Bayesian method of Zhivotovsky [44] implemented in *AFLP-SURV* v1.0 [45] with the option of non-uniform prior distributions of allele frequencies. In contrast with alternative procedures (e.g. Lynch and Milligan [46]), this Bayesian approach does not imply a pruning of loci with low frequency of null-alleles and produces statistically unbiased estimates of diversity and genetic distances [44]. Genetic diversity per sample was measured by assessing the number of polymorphic loci (5% criterion), Nei’s gene diversity and the presence of private bands. Significant differences in gene diversity between samples were tested with the T’ method for multiple unplanned comparisons between pairs of means based on equal sample sizes [47].

Allele frequencies were also used to estimate genetic differentiation between samples (*F*_ST_). *F*_ST_ values were calculated for the whole data set as well as for each area (exposed and sheltered coast) following Lynch and Milligan [46] and their significance tested with a permutation test (10,000 pseudoreplicates). Nei’s genetic distances between pairs of populations were used for UPGMA *(unweighted pair-group mean average)* cluster analysis and for Principal Coordinates Analysis (PCoA) with the help of MVSP v3.12d. (Kovach Computing Services) and GeneAlEx v6.1 [48] respectively. To facilitate comparison with other AFLP studies, differentiation was also estimated by the analysis of molecular variance (AMOVA, [49]) implemented in GeneAlEx v6.1. AMOVA calculates *Φ*_PT_, an analogue of *F*_ST_, using the squared Euclidean distance matrix between AFLP phenotypes and allows a hierarchical analysis of the genetic structures (e.g. differentiation between regions, between populations within regions). *Φ*_PT_ is a band-based approach that does not depend so critically on specific assumptions that could underestimate genetic variability [15], [49], [50] and has been specifically recommended for AFLP data [41].

The occurrence of isolation by distance (IBD) was explored with the Mantel test of correlation between the matrices of pairwise *Φ*_PT_ values and pairwise geographical distances (the shortest paths following the shore line between sites were measured with Google Earth). Both distances were used unprocessed and log-transformed in all possible combinations. Mantel test was performed with the online application *Isolation by Distance Web Service* [51].

In an alternative approach, the genetic structure was further investigated with the Bayesian model-based clustering algorithms implemented in STRUCTURE v2.3.3 [52]–[54] under a model assuming admixture and independent allele frequencies. Sampling locations were used as prior information to assist the clustering, as recommended when the signal of structure is weak. Ten runs with a burn-in period of 100,000 replications and a run length of 1,000,000 Markov chain Monte Carlo (MCMC) iterations were performed for a number of clusters (*K*) ranging 1–10. The *ad hoc* summary statistic Δ*K* of Evanno et al. [55] was used to select the value of *K* with the uppermost hierarchical level of population structure in our data. Anticipating that a superstructure might hide other structures at smaller spatial scales, STRUCTURE was run for the whole data set as well as for the populations within each area separately [55].

## Results

The six primer combinations produced a total of 230 AFLP loci, ranging from 29 to 52 per combination (Table S1), 99 (43%) of which were polymorphic (5% criterion) for the whole data set; every single individual produced a distinct multilocus genotype. The percentage of polymorphic loci per site was similar across sites except for S and CT, with the lowest and the highest values respectively (Table 1). Likewise, Nei’s gene diversity ranged from 0.123±0.010 in S to 0.224±0.011 in CT; these two extreme values were significantly different from estimates obtained at the other locations (*P*<0.01, test T’). Sites CH2 and LF showed one private band each, although with no evident link to any other parameter.

**Table 1:** *Nucella lapillus. S*ummary of AFLP markers and Nei’s gene diversity for every sample.

Site	N^a^	Polymorphic loci^b^	Nei’s gene diversity(± S.E.)	No. private bands
**Sheltered coast**				
Chazo2 (CH2)	29	93 (40.4%)	0.167±0.010	1
Chazo (CH)	30	89 (38.7%)	0.156±0.010	0
As Sinas (S)	30	68 (29.6%)	0.123±0.010	0
Arousa (A)	30	93 (40.4%)	0.174±0.011	0
Meloxo (M)	30	96 (41.7%)	0.182±0.010	0
**Exposed coast**				
Laxe_F (LF)	30	96 (41.7%)	0.194±0.011	1
Laxe_C (LC)	30	85 (37.0%)	0.158±0.010	0
St_Mariña (SM)	30	91 (39.6%)	0.165±0.010	0
C_Tosto (CT)	30	113 (49.1%)	0.224±0.011	0
Caldebarcos (C)	29	94 (40.9%)	0.186±0.011	0

a Average number of individuals takes into account the presence of missing data for some primer combinations.

b 5% criterion applied to Bayesian estimates of allele frequencies (Zhivotovsky 1999).

Both *F*_ST_ (0.172) and *Φ*_PT_ (0.254) statistics revealed the presence of highly significant genetic differences among sites *(P*<0.0001 and *P*<0.001 respectively) (Table 2); as expected, *Φ*_PT_ values exceeded those of *F*_ST_. Genetic differentiation was also significant when sheltered and exposed sites were considered separately but *F*_ST_/*Φ*_PT_ estimates were clearly higher for the complete data set than for each area, suggesting that genetic distances might follow a hierarchical structure linked to geographical distance. Despite comparable geographic distances within the two areas, genetic differentiation between exposed coast sites was twice as high as within the ria. Hierarchical AMOVA (Table 3) confirmed the occurrence of a hierarchical structure as differences between areas explained a higher fraction of the genetic variation (26%, *Φ*_RT_ = 0.258, *P*<0.001) than differences between populations within the same area (8%, *Φ*_PR_ = 0.110, *P*<0.001). Pairwise *Φ*_PT_ differentiation was always highly significant *(P*<0.01 after Bonferroni correction for multiple testing) and ranged from 0.021 for the comparison CH-CH2 (two sheltered sites separated 400 m) to 0.506 for SM-CH2 (exposed *vs.* sheltered site, over 100 km apart). Similarly, pairwise *F*_ST_ differentiation ranged from 0.014 for CH-CH2 to 0.405 for SM-S (Table 4).

**Table 2:** *Nucella lapillus.* Wright’s fixation index (*F*_ST_) and *Φ*_PT_ values.

	*F*_ST_ a	*Φ*_PT_ b
All populations (10)	0.172	0.254
	*P*<0.0001	*P*<0.001
Sheltered coast populations (5)	0.049	0.082
	*P*<0.0001	*P*<0.001
Exposed coast populations (5)	0.095	0.134
	*P*<0.0001	*P*<0.001

a Based on Bayesian estimates of allele frequencies (probability after 10,000 permutations).

b Calculated using Euclidean distances between AFLP phenotypes (probability after 1,000 permutations).

**Table 3:** *Nucella lapillus.* Hierarchical AMOVA with populations grouped in two areas (exposed and sheltered).

Source	df	SS	Estimated variance	*Φ* Statistics	*P* a
Between areas	1	871.2	5.38 (26%)	*Φ_RT = _*0.258	<0.001
Between populations	8	517.8	1.70 (8%)	*Φ_PR_* = 0.110	<0.001
Within populations	29	3,987.2	13.75 (66%)	*Φ_PT = _*0.340	<0.001

a Probabilities after 1,000 permutations.

**Table 4:** *Nucella lapillus.* Pairwise *F*_ST_ between populations (lower diagonal) and pairwise geographic distance in km (upper diagonal).

	Sheltered coast					Exposed coast				
	CH2	CH	S	A	M	C	CT	SM	LC	LF
**CH2**		0.370	4.280	6.800	13.240	57.830	101.390	106.510	115.370	115.430
**CH**	0.0139***		4.020	7.001	12.800	57.490	101.080	106.740	115.610	115.760
**S**	0.0576***	0.0434***		3.150	10.860	58.070	102.480	107.600	116.400	116.405
**A**	0.0368***	0.0390***	0.0540***		7.800	58.120	103.420	108.040	117.000	117.005
**M**	0.0569***	0.0749***	0.0806***	0.0308***		50.330	95.640	100.220	109.190	109.270
**C**	0.2223***	0.2132***	0.2228***	0.1500***	0.1332***		50.130	54.710	63.760	63.910
**CT**	0.1394***	0.1489***	0.1791***	0.0916***	0.0812***	0.0765***		5.000	13.700	13.780
**SM**	0.3698***	0.3810***	0.4046***	0.2983***	0.2579***	0.1219***	0.1426***		9.300	9.400
**LC**	0.3398***	0.3444***	0.3671***	0.2642***	0.2307***	0.1176***	0.1233***	0.0519***		0.800
**LF**	0.2253***	0.2190***	0.2405***	0.1494***	0.1392***	0.0638***	0.0611***	0.1149***	0.0747***	

*** *P*<0.001 (significance after 10,000 permutations).

**Figure 2:**
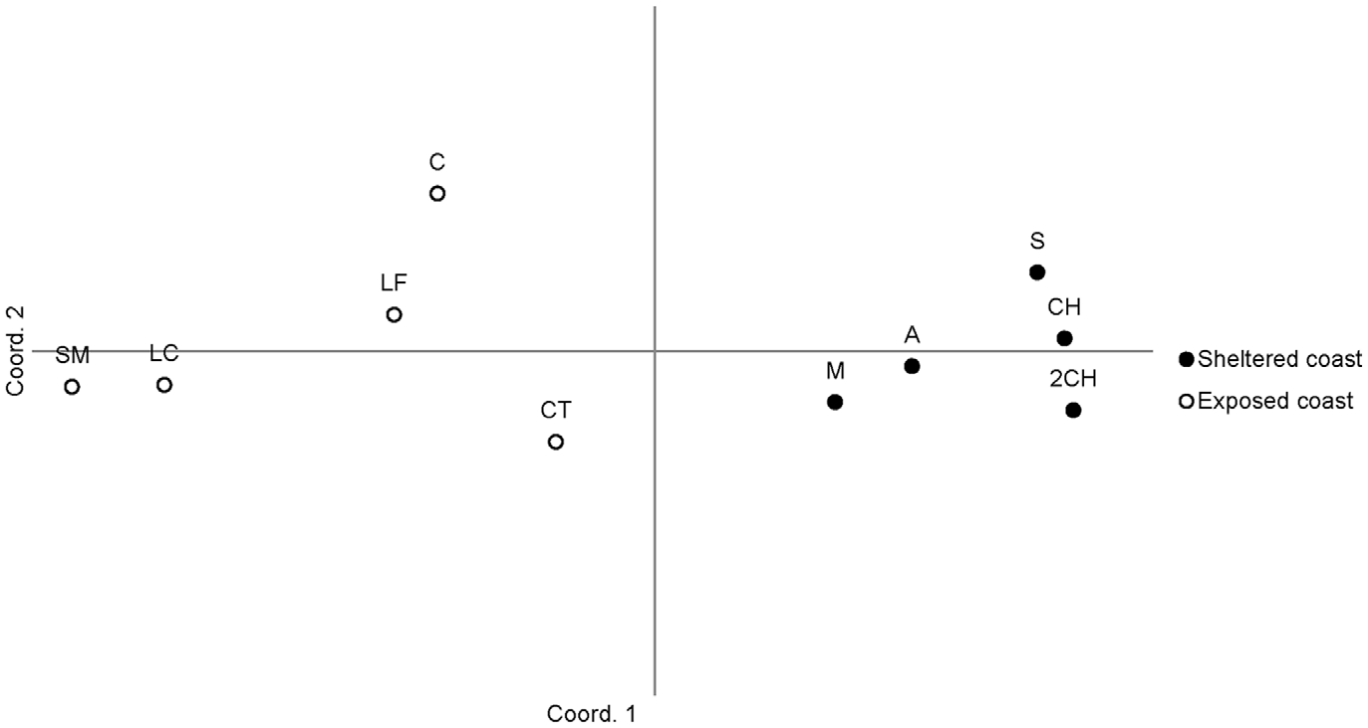
*Nucella lapillus.* Principal Coordinates Analysis. Nei’s distances between populations are used for the PCOA analysis. The first coordinate explains 79.9% of the variation; the second coordinate explains 6.4%.

The hierarchical structure was clearly illustrated both by the UPGMA cluster analysis (see Figure S1 in Supporting Information) and by the Principal Coordinates Analysis (PCoA, Figure 2) of Nei’s genetic distances between pairs of populations. Samples consistently formed two distinct, non-overlapping groups of exposed and sheltered coast sites. Separation between samples within each group was smaller than the distances found between samples from different areas. Furthermore, the position of sheltered coast sites within the PCoA ordination was highly consistent with their geographical arrangement. The same hierarchical structure could also be recognized when Euclidean genetic distances between individuals (not populations) were used to construct the PCoA plot (results not shown). Individuals from sheltered and exposed coast locations clearly separated into two compact groups along coordinate one. The only exceptions were (i) a small number of individuals collected in M (8) and A (2) that clustered with individuals from exposed coast sites, and (ii) 14 individuals from CT, four from C, and one from LF (exposed coast sites) that were plotted alongside with animals sampled within the ria.

**Figure 3:**
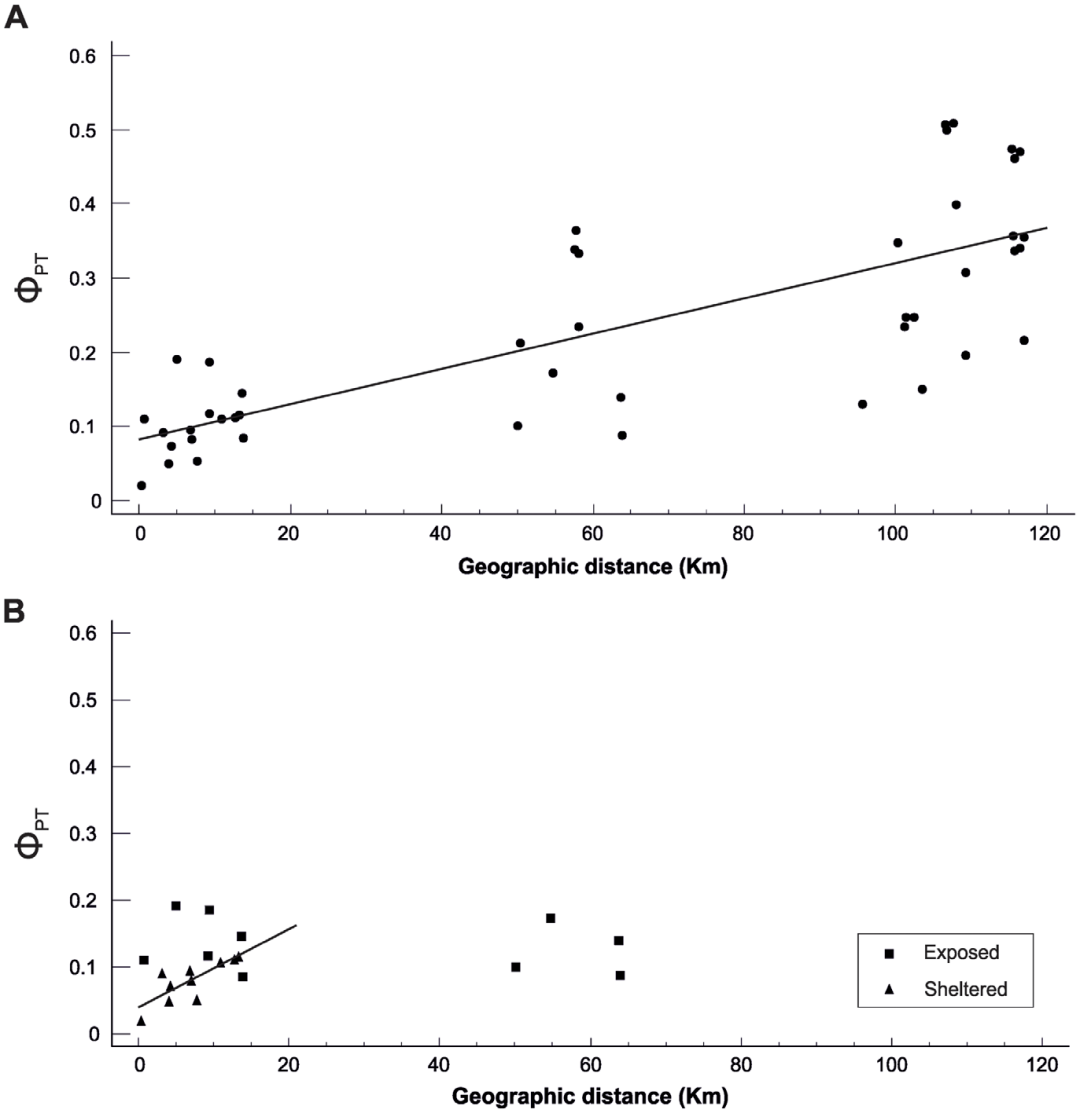
*Nucella lapillus.* Correlation analysis. Linear relationship between geographic and genetic distance between pairs of samples in A) all the sites (*r^2^* = 0.587, *P*<0.001) and B) in exposed and sheltered (*r^2^* = 0.644, *P*<0.001) coast separately. Line is the major axis regression between the two variables.

The occurrence of hierarchical structure suggested that *N. lapillus* might follow a model of isolation by distance. Mantel test confirmed this hypothesis (Figure 3) and the correlation between the logarithm of genetic distance and the logarithm of geographical distance was highly significant (reduced major axis regression: *Φ*_PT_ = 0.0330 *Dist* ^0^.^4911^, *r^2^* = 0.639, *P*<0.001 for 1,000 randomizations). The correlation was still evident when the test was restricted to sheltered coast sites (*Φ*_PT_ = 0.0317 *Dist* ^0^.^5030,^ *r^2^* = 0.773, *P*<0.051) but disappeared when only samples from exposed coast were considered in the analysis (*r^2^* = 0.014, *P*<0.43) (Figure 3).

Using the complete data set, STRUCTURE detected two clusters that matched the two studied areas (Figure 1). The cluster that drew together the sheltered coast sites showed some individuals with mixed ancestry. These individuals, which show a minor fraction of their membership belonging to the other cluster, were sampled within the two sites in the outermost part of the ria (M and A). In contrast, most of the individuals in the cluster corresponding to the exposed coast showed mixed ancestry except in SM (only one individual with mixed ancestry) and in CT (some individuals entirely assigned to the sheltered cluster). Separate analysis for sheltered coast sites returned identical results, whereas the analysis of the exposed coast revealed a genetic structure matching the sampling locations.

## Discussion

### Genetic Evidences of Low Dispersal in Nucella lapillus

The dogwhelk *Nucella lapillus* possesses attributes typical of a low dispersal organism. The absence of a planktonic larva together with a sedentary adult stage constrained to rocky intertidal enclaves are traits linked to limited dispersion [19], [21], [56]. In agreement with this, the conventional genetic analysis of our AFLP data (*F*_ST_, IBD) found a significant genetic differentiation among sites, indicating that *N. lapillus* does not form the single panmictic population expected in organisms with long-distance dispersal. Instead, genetic differentiation was strong between areas (sites separated by 50–120 km) as well as between sites within a single area (distances <15 km). Even sheltered sites separated as little as 400 m along an uninterrupted rocky intertidal suitable for *N. lapillus* showed significant genetic differences. Our *F*_ST_ estimates are comparable to those obtained for other gastropods with direct development [57]–[60]. Moreover, differentiation increased with geographical distance following an IBD pattern with a steep slope that suggests that dispersal must be low in this species. IBD was likewise evident at area scale within the sheltered ria, but not in the exposed coast where it was replaced by a seemingly unordered pattern.

The slopes of the isolation by distance lines can be grossly translated into estimates of mean dispersal distances using the theoretical model proposed by Kinlan and Gaines [4] based on an idea of Palumbi [61]. These estimates suggest that dogwhelks disperse, on average, between circa 300 meters per year (sheltered sites only) to 700 (exposed and sheltered sites analyzed together). From a compendium of genetic studies of marine organisms, Kinlan and Gaines [4] concluded that estimates of dispersal distances obtained for species lacking both planktonic larval phase and other secondary mechanisms of dispersion (as *N. lapillus*) typically were <<1 km whereas the average distances in organisms without planktonic phase but with secondary dispersal mechanisms (e.g. adults who are able to raft attached to floating structures, drift or have reproductive fragments) would reach some 10 Km. Therefore, the mean dispersal distances estimated for *N. lapillus* fall well within the typical range found in species with very low dispersal capacity. Our results are also in agreement with a pioneer allozyme work conducted on *N. lapillus* [62] where interpopulation variability, both for allele frequencies and heterozygosity, fitted the expectations for a species with direct development. Later, Goudet et al. [39] reanalyzed allozyme data finding overall F_ST_ estimates (0.3326) and a three level structure that provided further support to the low dispersal of the dogwhelks.

The low gene flow and restricted dispersal inferred with AFLP and allozyme data contrasts with the more recent proposal that dogwhelks must have larger dispersal ability and move longer distances than previously thought [31]. The reasons for this discrepancy are uncertain, but they may partly lie in the different approach of the data analysis used in each study. Colson and Hughes [31] based their proposal in the comparison of microsatellite genetic diversity and structure between continuous and recolonized sites after a partial ban on TBT antifouling paints imposed in 1987. Their finding that genetic diversity was non-significantly lower in recolonized populations was interpreted as evidence that recolonization of vacant sites had been accomplished by a relatively high number of individuals originating from several source populations. Later work based on quantitative genetic variation in shell form produced similar results [63]. However, genetic diversity is known to be notably resilient to reductions in effective population size and short bottlenecks must be very severe to have a substantial impact on heterozygosity. For example, conventional population genetics theory predicts that a bottleneck of size two still retains 75% of initial heterozygosity after one generation [64]. In this regard, the small declines in average gene diversity observed by Colson & Hughes (see Table 3 in [31]) in sites recolonized by *N. lapillus*, albeit statistically non-significant, are compatible with recolonization by a very low number of colonist (between 3 and 35) provided that census sizes were rapidly recovered after a short founding effect. Similarly, Piñeira et al. [65] did not find evidence of severe bottleneck effects (i.e. reductions in genetic variation) in populations of *Littorina saxatilis*, another marine gastropod with direct development, that had recovered from a massive oil spill.

### Wave Exposure and Population Structure

Our genetic markers reveal that, in addition to being genetically differentiated, our exposed and sheltered areas differed in the intensity and spatial pattern of their genetic structure. Exposed populations displayed stronger genetic differences among them but these differences did not conform to any spatial pattern. In comparison, populations inside the ria were genetically closer to each other but their differences fitted the expectations of an IBD pattern. Accordingly and after a hierarchical approach, STRUCTURE was still not able to resolve among sheltered IBD-patterned sites [66], but it did detect some structuring among the non-IBD patterned exposed ones. This variation in the genetic structure of exposed and sheltered areas is not unique to *N. lapillus*. The netted dogwhelk *Nassarius reticulatus* (L.) also showed a stronger genetic structure between exposed populations than between protected ones [37]. Therefore, our results seem consistent with the hypothesis that the environmental conditions of exposed sites may promote higher levels of genetic variability between local populations in both gastropods.

Despite these similarities, there are still interesting differences between *N. lapillus* and *N. reticulatus*. Specifically, the populations from inside the ria are genetically indistinguishable in *N. reticulatus* while they retain significant levels of genetic differentiation in *N. lapillus*. Since both gastropods were studied in the same geographical context (NW Spain), it seems reasonable to presume that these differences in the intensity of the genetic structure in sheltered sites are possibly linked to the contrasting dispersal ability of each species. Thus, while veligers of *N. reticulatus* spend 1–2 months in the plankton and its adults are very mobile [67]–[69], *N. lapillus* is a snail with direct development and a reputation for very sedentary adults (see Fretter and Graham [70] and references therein). Hence, the stronger genetic structure and IBD pattern found inside the ria for *N. lapillus* seems consistent with its life history traits and provides further support to the conclusion that this dogwhelk must disperse little, at least in sheltered areas with low hydrodynamics.

Part of the genetic differences detected inside the ria is due to a gradual increase of individuals with a distinct genetic composition towards its mouth. Both PCoA and STRUCTURE reveal that the two enclaves located in the lower reaches of the ria (M, A) are genetically closer to open-coast populations than those from the upper reaches (S, CH, CH2). STRUCTURE showed that the admixture proportion is higher in the individuals sampled in site M (closer to the entrance of the ria) which genetic composition assigned to the cluster dominating in exposed populations. The origins of the gradual increase of a distinct genetic composition towards the mouth of the ria are uncertain. Two hypotheses can be invoked to explain the admixture proportion found in individuals in the mouth of the ria. One explanation is that a fraction of the genome could have been inherited from a migrant ancestor. Alternatively, the occurrence of dogwhelks from two genetic clusters within the same location has formerly been related to the existence of individuals belonging to two different shell morphotypes (exposed and sheltered) [71]. Although sheltered and exposed morphs are typically found at localities with contrasting levels of exposure to wave action, they also occur sympatrically in some NW Spain sites [71], [72]. In the latter, Guerra-Varela et al. [71] found significant microsatellite differentiation between the two morphs and each morphotype was mostly assigned to a different genetic cluster by STRUCTURE. Nevertheless, their results show that dogwhelks from localities with moderate protection comparable to our M and A sites are also divided into the two genetic groups even though they all share the sheltered morph.

The situation is very different in exposed populations. Their unordered arrangement of genetic differences resembles the structure found in other coastal organisms studied at small-to-moderate spatial scales [14], [18], [37], [73], [74] and adds to the growing body of evidence that suggests that geographic distance is often a poor predictor of gene flow in coastal systems [75]. Traditionally, this “chaotic genetic patchiness” [76] has been interpreted as evidence that larval pools and recruitment are heterogeneous, and several mechanisms (limited mixing of larvae from distinct sources, patchy environmental selection, sweepstakes reproductive success, or even a combination of local recruitment with low dispersion) have been proposed to explain this heterogeneity (see Selkoe et al. [14] and references therein). However, recent studies suggest that the genetic patterns of coastal organisms possibly reflect more than just larval dispersal. Hence, habitat factors (e.g. size of suitable habitat) have been found to be more informative predictors of the genetics for different species than oceanographic features (temperature, currents, distance) suggesting that the genetic structure may be driven predominantly by variations in effective population sizes (*N_e_*) instead of migration rates (*m*) [18]. Further work is needed to corroborate whether these conclusions apply to other coastal systems. Yet, the proposal that the genetic structure might not be tightly linked to dispersal among populations would explain why organisms with substantial differences in their potential to disperse (e.g. *N. lapillus* in this study *vs. N. reticulatus* in Barreiro et al. [37]) manage to develop a similarly unordered pattern along wave-exposed coasts (for other examples of discrepancies between life traits and genetic differentiation see Galarza et al. [17] and references therein).

Most efforts to explain chaotic genetic patchiness have focused on recruitment from a larval pool, sometimes from distant sources, since this pattern is mostly detected in organisms with planktonic larvae. However, dogwhelks are direct-developers and our results suggest that dispersal is restricted, at least under some conditions. If limited dispersal were also applicable to wave-exposed populations, they should be mostly maintained by self-recruitment. Under this hypothesis, we would predict a spatially stable pattern of genetic differences over time and/or across age groups [77]. Alternatively, if stronger wave action enhances dispersal, exposed populations would be maintained by both self-recruits and immigrants resulting in spatio-temporal changes in the genetic structure. Finally, spatial structure may depend on variations of *N_e_* rather than *m* as proposed by Selkoe et al. [18]. We are not aware of any studies on changes of *N_e_* over time in *N. lapillus*. However, some long-term monitoring surveys suggest that dogwhelks may experience appreciable changes in local abundance that, more importantly, may not be synchronous between sites [78]. Should these changes have an impact on *N_e_*, we would anticipate variations in the spatial genetic structure over time. Future work comparing population structure across age groups to test this prediction seems warranted.

In summary, using a sizeable number of reproducible AFLP markers, we found significant interpopulation differentiation at both regional (<200 km) and areal (<15 km) scales. Genetic differences followed an IBD pattern at regional scale as well as among sheltered populations within a ria, indicative of restrictions to gene flow. Thus, our results support the conventional view of *N. lapillus* as a poor disperser and seem consistent with earlier allozyme studies as well as with predictions derived from its life history features [7], [38], [39], [62], [70], [79]. Nevertheless, the variation in the genetic arrangement observed within the two areas (sheltered and exposed) analyzed in this study seems consistent with an impact of the hydrodynamic regime on the spatial genetic structure. The correlation between spatial and genetic distances detected among our sheltered sites vanished in wave-exposed populations. The latter showed the unordered arrangement of genetic distances typical of other coastal organisms. The mechanisms behind this unordered patchiness are not clear and further work is required to unravel them.

## Supporting Information

Figure S1 ***Nucella lapillus.*** **UPGMA analysis based on Nei’s genetic distances between sites.**
(TIF)

Table S1 **Primer sequences used for the AFLP selective amplification and number of loci generated.**
(DOCX)
