# Supplementary figures and images for: AFLPs Reveal Different Population Genetic Structure under Contrasting Environments in the Marine Snail Nucella lapillus L
Source: PLoS One. 2012 Nov 21;7(11):e49776. doi: 10.1371/journal.pone.0049776 (PMC3504068; doi:10.1371/journal.pone.0049776)

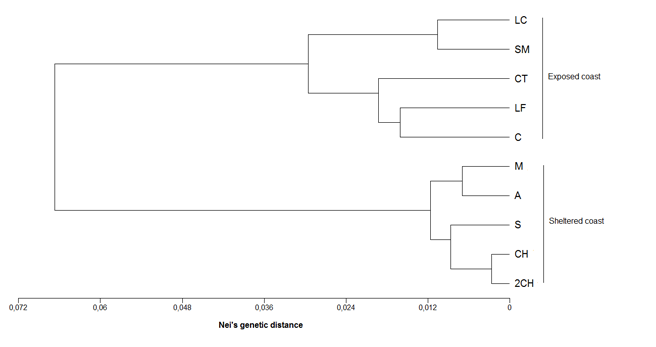

Supplement: Figure S1 — Nucella lapillus. UPGMA analysis based on Nei’s genetic distances between sites. (TIF) [file pone.0049776.s001.tif]
