# Supplementary material for: AFLPs Reveal Different Population Genetic Structure under Contrasting Environments in the Marine Snail Nucella lapillus L
Source: PLoS One. 2012 Nov 21;7(11):e49776. doi: 10.1371/journal.pone.0049776 (PMC3504068; doi:10.1371/journal.pone.0049776)
Supplement: Table S1 — Primer sequences used for the AFLP selective amplification and number of loci generated. (DOCX) [file pone.0049776.s002.docx]

**Table S1. Primer sequences used for the AFLP selective amplification and number of loci generated.**

| Primer combination | *EcoRI-*primer (5’-3’) | *Tru1I-*primer (5’-3’) | No. of loci |
| --- | --- | --- | --- |
| 1 | gactgcgtaccaattc+tAG | gatgagtcctgagtaa+cGT | 52 |
| 2 | gactgcgtaccaattc+tAG | gatgagtcctgagtaa+cAC | 40 |
| 3 | gactgcgtaccaattc+tAG | gatgagtcctgagtaa+cTG | 29 |
| 4 | gactgcgtaccaattc+tCT | gatgagtcctgagtaa+cGT | 34 |
| 5 | gactgcgtaccaattc+tCT | gatgagtcctgagtaa+cAC | 38 |
| 6 | gactgcgtaccaattc+tCT | gatgagtcctgagtaa+cTA | 37 |
